# Insulin Therapy of Nondiabetic Septic Patients Is Predicted by *para*-Tyrosine/Phenylalanine Ratio and by Hydroxyl Radical-Derived Products of Phenylalanine

**DOI:** 10.1155/2015/839748

**Published:** 2015-10-20

**Authors:** Szilárd Kun, Gergő A. Molnár, Eszter Sélley, Lívia Szélig, Lajos Bogár, Csaba Csontos, Attila Miseta, István Wittmann

**Affiliations:** ^1^2nd Department of Medicine and Nephrological Centre, Faculty of Medicine, University of Pécs, Pacsirta Street 1, Pécs 7624, Hungary; ^2^Department of Anaesthesia and Intensive Care, Faculty of Medicine, University of Pécs, Rákóczi Street 2, Pécs 7623, Hungary; ^3^Department of Laboratory Medicine, Faculty of Medicine, University of Pécs, Ifjúság Street 13, Pécs 7624, Hungary

## Abstract

Hydroxyl radical converts Phe to *para*-, *meta*-, and *ortho*-Tyr (*p*-Tyr, *m*-Tyr, *o*-Tyr), while Phe is converted enzymatically to *p*-Tyr in the kidney and could serve as substrate for gluconeogenesis. Pathological isoforms *m*- and *o*-Tyr are supposed to be involved in development of hormone resistances. Role of Phe and the three Tyr isoforms in influencing insulin need was examined in 25 nondiabetic septic patients. Daily insulin dose (DID) and insulin-glucose product (IGP) were calculated. Serum and urinary levels of Phe and Tyr isoforms were determined using a rpHPLC-method. Urinary *m*-Tyr/*p*-Tyr ratio was higher in patients with DID and IGP over median compared to those below median (*P* = 0.005 and *P* = 0.01, resp.). Urinary *m*-Tyr and *m*-Tyr/*p*-Tyr ratio showed positive correlation with DID (*P* = 0.009 and *P* = 0.023, resp.) and with IGP (*P* = 0.004 and *P* = 0.008, resp.). Serum Phe was a negative predictor, while serum *p*-Tyr/Phe ratio was positive predictor of both DID and IGP. Urinary *m*-Tyr and urinary *m*-Tyr/*p*-Tyr, *o*-Tyr/*p*-Tyr, and (*m*-Tyr+*o*-Tyr)/*p*-Tyr ratios were positive predictors of both DID and IGP. Phe and Tyr isoforms have a predictive role in carbohydrate metabolism of nondiabetic septic patients. Phe may serve as substrate for renal gluconeogenesis via enzymatically produced *p*-Tyr, while hydroxyl radical derived Phe products may interfere with insulin action.

## 1. Introduction

Kidney has an important role in carbohydrate metabolism. There are evidences that renal glucose release contributes to a significant proportion in maintaining fasting plasma glucose level (~25%) [1, 2]. As the length of fasting increases, the proportion of overall glucose release accounted for by renal gluconeogenesis increases [3]. Releasing glucose from the kidney is solely the result of gluconeogenesis [2]. In type 2 diabetic humans, the rate of renal glucose release is increased in a large extent (~300%) and it becomes comparable with hepatic glucose release [4]. Also in hyperepinephrinemia, kidney is responsible for approximately 40% of increased gluconeogenesis [5]. Renal glucose release is inhibited by insulin. This inhibitory effect involves both direct activation or deactivation of enzymes and lowered availability of gluconeogenic substrates [6, 7].

Renal gluconeogenesis is connected to Phe and Tyr metabolism, since phenylalanine hydroxylase, which converts Phe to* para*-Tyr (*p*-Tyr), is located in renal epithelial cells [8] and since Tyr can serve as a substrate for gluconeogenesis through its metabolites, as fumarate [9]. Sepsis is described as a catabolic state with overproduction of endogenous cortisol with increased gluconeogenesis [10]. Moreover, patients with septic shock are often requiring intravenous administration of hydrocortisol, which has a contrainsular effect. Glucose homeostasis in sepsis is also a complex issue, involving endogenous glucose production, endogenous insulin production, and exogenous substitution of either glucose or insulin.

A hallmark of sepsis is oxidative stress. In former studies of our workgroup, we found elevated levels of malondialdehyde (MDA) and myeloperoxidase (MPO) in the early phase of sepsis. There was an increased production of phorbol 12-myristate 13-acetate (PMA) which stimulated reactive oxygen species (ROS) in whole blood on first and second days in septic patients [11]. In the study of Ware et al. higher levels of lipid peroxidation products like plasma F2-isoprostane and isofuran were found in septic patients when organ failure developed [12].

The resulting reactive oxygen species may exert damage among others to amino acids. Overproduction of hydroxyl radical (HO^∙^) converts Phe into* para*-,* meta*-, and* ortho*-Tyr (*p*-,* m*-, and* o*-Tyr) [13, 14]. On the other hand, export* p*-Tyr (which is used by other organs for protein synthesis) is formed enzymatically from Phe under physiological circumstances in the kidney, through Phe hydroxylase, as mentioned above [15]. Thus,* p*-Tyr may be formed physiologically and in the oxidative processes as well, while* m*- and* o*-Tyr are oxidative stress markers only. Consequently, elevated levels of* m*- and* o*-Tyr detect hydroxyl radical-induced tissue damage. Many studies have proved that* m*- and* o*-Tyr levels correlated with other oxidative stress markers [16–18].

Increasing number of studies assessed serum and urinary Tyr isoforms in different illnesses. Serum and urinary* o*- and* p*-Tyr levels were measured by our group in patients suffering from diabetes mellitus and chronic kidney disease (CKD). Significantly lower plasma* p*-Tyr levels were found in CKD group, while increased urinary excretion of* o*-Tyr was observed in diabetic/CKD patients [19]. Interestingly, total urinary albumin/creatinine and nonimmunoreactive albumin/creatinine ratios showed a good correlation with urinary* o*-Tyr/creatinine ratio in patients suffering from ischemic stroke in our other study [20]. Also, we found higher levels of* m*-Tyr and* o*-Tyr in the total homogenates of cataractous lenses [21]. These pathological Tyr isoforms may have a role in development of hormone resistances, as insulin or erythropoietin resistance. This has recently been proved by our group in both* in vitro* and human studies [22–24].

Based on the previous data we postulated that elevated levels of hydroxyl radical-derived Tyr isoforms along with enzymatically produced* p*-Tyr could contribute to altered carbohydrate metabolism in nondiabetic septic subjects and thus predict insulin therapy of these patients. Phe and* p*-Tyr contribute to renal gluconeogenesis while pathological isoforms (*m*- and* o*-Tyr) may interfere with insulin action in the kidney, thus leading to insulin resistance.

## 2. Methods

### 2.1. Subjects and Study Design

The study protocol was approved by the Ethical Committee of the Medical Faculty of the University of Pécs (4422/2012) and it was completed in accordance with the ethical guidelines of the 2003 Declaration of Helsinki. All the patients or the nearest relatives provided a written informed consent after enlightenment. The study was performed on 25 patients admitted to the Department of Anaesthesia and Intensive Care, Faculty of Medicine, University of Pécs, between September 2012 and October 2013. Those patients who presented with severe sepsis or septic shock at admission were included in the study. The diagnosis of sepsis was based on the ACCP/SCCM consensus guideline [25].

Exclusion criteria were medication (e.g., chronic steroid use and immunosuppressive medication) or treatment (e.g., radio- and chemotherapy) affecting the normal immune response and hematologic malignant disease and oliguria at admission (collection of urine samples was impossible). Patients were treated according to the recent sepsis guideline [26]. Blood samples were taken on admission (day 1) and on the four consecutive days (days 2–5). Urine has been collected every 24 hours and the daily amount has been noticed.

A five-day long study period has been chosen because we had presumed that a 5-day period would open a wide time window that could be enough for detecting early changes in* p*-,* m*-, and* o*-Tyr and Phe levels in patients suffering from sepsis. Serum and urinary creatinine, serum hsCRP, and PCT levels were measured. Daily hsCRP, PCT, and creatinine measurements were part of the routine monitoring of septic patients and they were carried out at the Institute of Laboratory Medicine, University of Pécs.

Patients received insulin intravenously using perfusor according to a sliding-scale to maintain blood glucose level in the range of 6–8 mmol/L. Glucose levels were measured using arterial blood gas analysis at minimum 5 times per day. Data of daily insulin dose (DID) and glucose profile were assessed. An insulin-glucose product (IGP) was calculated based on DID and mean daily glucose levels.

In case of septic shock, after fluid resuscitation (20 mL/kg crystalloid solution) hydrocortisone (200 mg/24 h) and norepinephrine were administered to maintain MAP > 70 mmHg. Invasive haemodynamic monitoring was started too. If central venous saturation of hemoglobin (ScVO2) remained lower than 70% and cardiac index (CI) was below 2.5 L/min/m^2^ despite adequate preload (intrathoracic blood volume index (ITBVI)) dobutamine was added to the treatment. Daily doses of these agents were also noticed.

### 2.2. Measurement of* p*-,* m*-, and* o*-Tyr and Phe Levels

**Figure 1:**
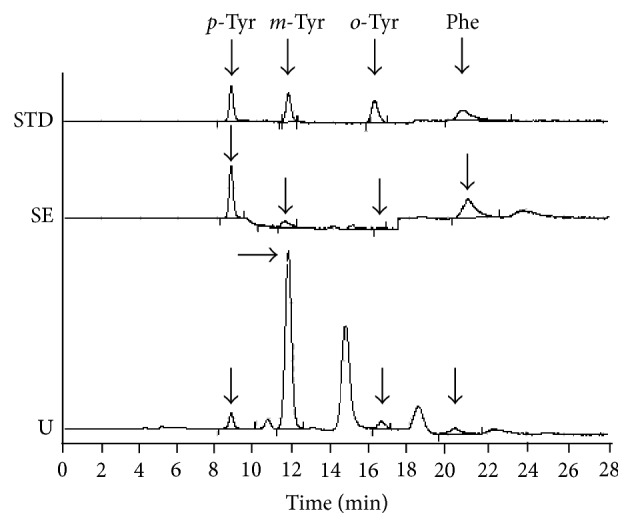
Chromatograms of a standard (STD), a serum (SE), and a urine sample of a septic patient (U).* p*-Tyr,* para*-tyrosine;* m*-Tyr,* meta*-tyrosine;* o*-Tyr,* ortho*-tyrosine; Phe, phenylalanine.

Blood samples of septic patients were obtained from a central venous catheter. Serum was obtained by centrifugation. Serum and urine samples were stored at −80°C until further examinations. Thereafter, 125 *µ*L trichloroacetic acid (TCA; Reanal Private Ltd., Budapest, Hungary) was added to 500 *µ*L serum or urine and then samples were incubated on ice for 30 min. Subsequently precipitate was separated by centrifugation. The supernatant was filtered by a syringe filter (0.2 *µ*m) (Millipore, Billerica, MA, USA) before analysis. Finally serum and urinary* m*-,* o*-, and* p*-Tyr and Phe levels were determined using reverse phase-HPLC (Shimadzu USA Manufacturing INC, Canby, OR, USA) (C_18_ silica column, 250 × 4 mm) with fluorescence detection (*λ*_EX_ = 275 nm; *λ*_EM_ = 305 nm for the tyrosines and *λ*_EX_ = 258 nm; *λ*_EM_ = 288 nm for Phe) as described earlier [19]. Concentrations were calculated using an external standard. Representative HPLC profiles of a standard and of serum and urine samples of a septic patient are presented in Figure 1.

Fractional excretions (FE) of the three Tyr isoforms were calculated (FE_*p*-Tyr_, FE_*m*-Tyr_, and FE_*o*-Tyr_) to determine tubular handling of them. FE of a certain substance can be used to examine renal handling of that particular substance. It is calculated by dividing the clearance of the measured substance by the clearance of creatinine. FE shows how much of the filtered substance is excreted with the urine. It therefore indicates whether a clearance of the particular substance is greater or smaller than or equal to the clearance of creatinine. If FE of a substance is 100%, it is freely filtered, and the net renal reabsorption and secretion are zero. If FE is smaller than 100%, it indicates an active renal reabsorption of the substance. If FE exceeds 100%, it indicates active secretion or* in loco* renal production of the substance. For calculating 24-hour clearance and FE the respective blood sample has to be obtained during the urine collection.

### 2.3. Statistical Analysis

Statistical Package for the Social Sciences (SPSS) Statistics software, version 20.0 (IBM Corporation, USA), was used for statistical analysis. Data were expressed as median, interquartile range (IQR (standard 25th–75th percentile)) and whiskers represent 5th and 95th percentiles since their distribution was not normal by Kolmogorov-Smirnov test. Intergroup analyses were performed using Mann-Whitney *U* test. Correlations between variables were assessed using Spearman's rho test. Multivariate linear regression models with stepwise method were used to determine predictors of insulin demand. Values of *P* < 0.05 were considered significant.

## 3. Results

### 3.1. Demographic Data of Patients

Twenty-five septic patients were involved in the study. Demographic data, source of infections, and amino acid parameters are summarized in Table 1. Eight patients suffered from severe sepsis and 17 from septic shock and 19 patients required mechanical ventilation during ICU stay. Out of 25 patients, 11 were discharged from the ICU, while 7 patients died during the study period and 7 patients thereafter.

We examined the association of amino acid parameters and clinical outcomes. None of these metabolites showed any difference between survivor and nonsurvivor subjects on day 1 (data not shown).

### 3.2. Urinary* m*-Tyr Levels and Insulin Demand

**Figure 2:**
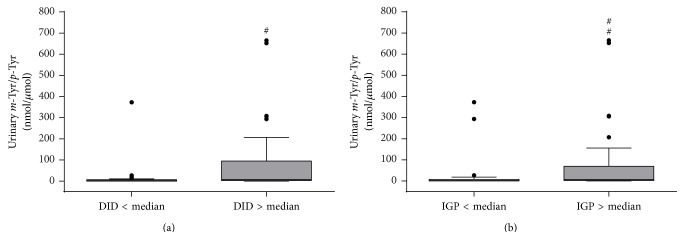
Urinary* m*-Tyr/*p*-Tyr ratio in septic patients requiring insulin administration, according to (a) daily insulin dose or (b) insulin-glucose product. ^#^ *P* = 0.005 versus DID < median; ^##^ *P* = 0.01 versus IGP < median. DID, daily insulin dose; IGP, insulin-glucose product.

Urinary* m*-Tyr/*p*-Tyr ratio was significantly higher in patients with DID over median compared to those with DID below median (7.3 92.3 versus 1.7 6.3, resp.; *P* = 0.005) (Figure 2(a)). Similarly, urinary* m*-Tyr/*p*-Tyr ratio was significantly higher in patients with IGP over median compared to those with IGP below median (6.6 67.5 versus 1.7 7.5, resp.; *P* = 0.010) (Figure 2(b)).

### 3.3. Association of Tyr Parameters with Insulin Demand

**Figure 3:**
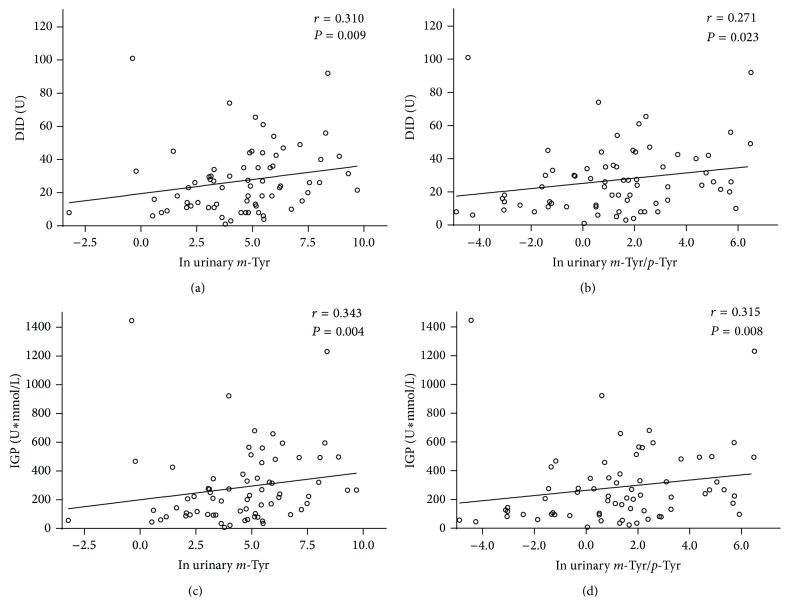
Correlation of urinary* m*-Tyr concentration with (a) DID and (c) IGP. Correlation of urinary* m*-Tyr/*p*-Tyr ratio with (b) DID and (d) IGP in septic patients requiring insulin administration. DID, daily insulin dose; IGP, insulin-glucose product.

Urinary* m*-Tyr concentration showed a positive correlation with DID (*r* = 0.310; *P* = 0.009) (Figure 3(a)) and with IGP (*r* = 0.343; *P* = 0.004) (Figure 3(c)). Similarly, urinary* m*-Tyr/*p*-Tyr ratio showed a positive correlation with DID (*r* = 0.271; *P* = 0.023) (Figure 3(b)) and with IGP (*r* = 0.315; *P* = 0.008) (Figure 3(d)).

Amino acid parameters were tested separately in multivariate linear regression models as predictors of insulin demand (DID and IGP). The components of this model were body weight, hsCRP, PCT, daily hydrocortisone dose, and daily dobutamine dose. Serum Phe was a negative predictor of both DID and IGP, while serum* p*-Tyr/Phe ratio associated positively and strongly with these carbohydrate metabolism parameters. Serum* o*-Tyr/Phe ratio was a positive predictor of IGP only, but not of DID (Table 2). Urinary level of* m*-Tyr and ratios of urinary* m*-Tyr/*p*-Tyr,* o*-Tyr/*p*-Tyr, and (*m*-Tyr+*o*-Tyr)/*p*-Tyr were positive predictors of both DID and IGP (Table 2). The abovementioned amino acid parameters were tested also in another model, in which serum creatinine level was included instead of body weight. In this model, similar results were obtained compared to the case of body weight (data not shown).

### 3.4. Fractional Excretion of Tyr Isoforms

Both FE_*m*-Tyr_ and FE_*o*-Tyr_ were significantly higher than FE_*p*-Tyr_ (*P* < 0.001, for both). There was also a significant difference between FE_*m*-Tyr_ and FE_*o*-Tyr_, as FE_*o*-Tyr_ was more than threefold higher than FE_*m*-Tyr_ (*P* = 0.006) (Table 1). No direct connection between FE values of any of the investigated amino acid and carbohydrate parameters was found.

## 4. Discussion

In our study, we provided evidence that elevated levels of hydroxyl radical-derived Tyr isoforms along with enzymatically produced* p*-Tyr could contribute to altered carbohydrate metabolism in nondiabetic septic subjects and thus predict insulin therapy of these patients.

Serum and urinary levels and ratios of the abovementioned amino acids were strong predictors of both DID and IGP in a model which was composed by the known predictors of elevated insulin demand. In another model, with serum creatinine level instead of body weight, the same results were obtained, indicating that these associations are independent of renal function.

A potential cause of the abovementioned association of serum level of Phe with DID and with IGP could be a generalized hypoaminoacidemia due to malnutrition. We tested this possibility by performing correlation tests between serum level of albumin and DID or IGP. Neither DID nor IGP showed correlation with serum albumin level (*r* = 0.056, *P* = 0.646; *r* = 0.048, *P* = 0.693, resp.) (data not shown).

The fact that (i) serum level of Phe proved to be a negative predictor of DID and IGP and (ii) serum* p*-Tyr/Phe ratio proved to be a stronger positive one, while (iii) serum* p*-Tyr alone was not a predictor and (iv) serum* p*-Tyr level was slightly but not significantly lower in septic patients compared to that of healthy subjects [19, 23] may suggest that in septic patients the conversion of Phe to* p*-Tyr—at a normal Phe level—results not only in the production of export* p*-Tyr, but the produced* p*-Tyr is also consumed for gluconeogenesis* in loco* in the kidney.

We are aware that, beyond oxidative stress, also other factors (such as metabolic status, actual level of inflammation, anthropometric parameters, and medications) may also have a strong influence on glycemic control. That is why we subsequently performed linear regression analyses, where correction to body weight, inflammatory markers, and doses of gluconeogenesis-stimulating agents did not lead to a disappearance of the association between* m*-Tyr and markers of glycemic status. Indeed, in this analysis, a statistically highly significant connection was observed. This suggests that, besides already known parameters, independently of them, the oxidative stress-derived amino acid does play a role in determining carbohydrate control.

Furthermore, our aim was mainly not to establish a new daily clinical routine marker for managing individual septic patients, but rather to enlighten the underlying mechanisms of carbohydrate metabolism of these patients in light of Phe and Tyr metabolism. However, we also think that these metabolites could be used as clinical markers as well, if they are considered together with other current routine clinical parameters, also mentioned in our study. As in many other cases, individual consideration will be the right way also in this certain issue.

There was a significant difference between FE_*m*-Tyr_ and FE_*o*-Tyr_. FE_*o*-Tyr_ was more than threefold higher than FE_*m*-Tyr_. At the same time serum concentrations of the two Tyr isoforms were equal. This could indicate that renal retention of* m*-Tyr is much higher than that of* o*-Tyr, which results in a higher intracellular concentration of* m*-Tyr in renal cells. This could explain better predictive role of* m*-Tyr.

A septic model has been chosen because rapid changes occur in carbohydrate metabolism and also serum and urinary levels of amino acids can be well monitored in these circumstances. Also short-term associations can be observed between carbohydrate metabolism and these amino acids.

In our study, the investigated parameters did not show any difference between groups based on ICU survival (data not shown). However, in the comparison of ICU-surviving versus nonsurviving patients, the number of cases in the two groups was only *n* = 18 versus *n* = 7. The low number of cases could be in part in the background of the lack of significance; the absolute values seemed to show a tendency that corresponds to our hypothesis; that is, markers of oxidative stress-derived amino acids seemed somewhat higher in the nonsurvival than in the survival group.

IGP in describing carbohydrate metabolism was used as a parameter referring to insulin resistance. This is similar to HOMA_IR_ in which plasma levels of fasting glucose and fasting endogenous insulin are included. If plasma level of glucose increases at a constant plasma level of endogenous insulin or vice versa the product rises, that refers to insulin resistance. The same conception was applied in case of IGP, in which the amount of exogenously administered insulin was multiplied with average plasma glucose level. On the other hand, according to our opinion, insulin need (thus DID and IGP) is predicted also by gluconeogenesis, as also endogenously produced glucose needs to be overcome by exogenously administered insulin. Furthermore, on the routine, no exogenous glucose infusion was applied in these patients, making the estimation of glucose metabolism somewhat easier. Thus we believe that DID and IGP mainly serve as descriptors of insulin resistance + gluconeogenesis.

A five-day long study period is appropriate for better observing the role of kidney, as in this duration renal glucose release contributes to total glucose release in an increasing proportion, as hepatic glucose output decreases, because of lowered glycogenolysis [3].

Aromatic amino acids, as Phe and Tyr, were assessed as predictors of glycemia cross-sectionally in a recent study in young adults. Serum Phe was positively associated with HOMA-IR in both males and females, while Tyr was only predictor of HOMA-IR in men [27].

Limitations of our study are the fact that (i) this is a pilot study with a relative low number of cases, (ii) correlational and linear regression analyses at each day separately were lacking, due to relatively low number of cases, and (iii) effect of hydroxyl radical-derived Phe products on insulin action in the kidney has not been proved directly.

## 5. Conclusions

Concluding, we provided evidence that Phe and its hydroxyl radical-derived products, along with enzymatically produced* p*-Tyr, predict insulin therapy of nondiabetic septic patients, which reflects (i) the role of kidney in gluconeogenesis, (ii) involvement of Phe and* p*-Tyr in gluconeogenesis, and (iii) the possible inhibitory effect of hydroxyl radical-derived Phe products (*m*- and* o*-Tyr) on insulin action.

## Figures and Tables

**Table 1:** Baseline demographics, clinical data, and amino acid parameters of patients.

*n*	25
Age [years]	69 ± 14
Male/female	14/11
Body weight [kg]	80 (13)
Source of sepsis	
Lung	13
Kidney	2
Burned skin	7
Wound	1
Abdomen	2
APACHE II	16 (10)
MODS	5 (5)
SAPS II	37.5 (20)
Serum creatinine [*µ*mol/L]	118 95.5
hsCRP [mg/L]	154.2 ± 92.8
PCT [ng/mL]	7.62 19.23
Daily urine output [mL]	2055 (1775)
Mean daily glucose [mmol/L]	9.0 2.5
DID [U/day]	19 (25)
IGP [U∗mmol/L]	183.4223 261.2992
Number of patients receiving hydrocortisone	18 (72%)
Daily hydrocortisone dose [mg/day]	200 (100)
Number of patients receiving dobutamine	10 (40%)
Daily dobutamine dose [mg/day]	275.0 537.5
Serum *p*-Tyr [*µ*mol/L]	45.177 ± 18.049
Serum *m*-Tyr [nmol/L]	14 (27)
Serum *o*-Tyr [nmol/L]	14 (15)
Serum Phe [*µ*mol/L]	65.374 ± 27.519
Serum *p*-Tyr/Phe [*µ*mol/*µ*mol]	0.654 0.292
Serum *m*-Tyr/Phe [nmol/*µ*mol]	0.2 0.4
Serum *o*-Tyr/Phe [nmol/*µ*mol]	0.2 0.4
Serum *m*-Tyr/*p*-Tyr [nmol/*µ*mol]	0.3 0.5
Serum *o*-Tyr/*p*-Tyr [nmol/*µ*mol]	0.3 0.5
Serum (*m*-Tyr+*o*-Tyr)/Phe [nmol/*µ*mol]	0.5 0.7
Serum (*m*-Tyr+*o*-Tyr)/*p*-Tyr [nmol/*µ*mol]	0.6 1.1
Urinary *p*-Tyr [*µ*mol/L]	25.394 44.402
Urinary *m*-Tyr [nmol/L]	123 (351)
Urinary *o*-Tyr [nmol/L]	194 (661)
Urinary *m*-Tyr/*p*-Tyr [nmol/*µ*mol]	4 (17)
Urinary *o*-Tyr/*p*-Tyr [nmol/*µ*mol]	9 (39)
Urinary (*m*-Tyr+*o*-Tyr)/*p*-Tyr [nmol/*µ*mol]	21 (85)
Urinary *p*-Tyr/creatinine [*µ*mol/mmol]	6.952 11.645
Urinary *m*-Tyr/creatinine [nmol/mmol]	29 (68)
Urinary *o*-Tyr/creatinine [nmol/mmol]	61 (220)
Daily excretion of *p*-Tyr [*µ*mol/day]	56.268 116.710
Daily excretion of *m*-Tyr [nmol/day]	259 (607)
Daily excretion of *o*-Tyr [nmol/day]	304 (1786)
Clearance of *p*-Tyr [mL/min]	0.605 1.720
Clearance of *m*-Tyr [mL/min]	9.011 27.413^*^
Clearance of *o*-Tyr [mL/min]	17.198 105.512^∗†^
FE_*p*-Tyr_ [%]	1.783 2.160
FE_*m*-Tyr_ [%]	25.104 54.305^‡^
FE_*o*-Tyr_ [%]	85.645 639.219^‡§^

^*^ *P* < 0.001 versus clearance of *p*-Tyr; ^†^ *P* = 0.019 versus clearance of *m*-Tyr; ^‡^ *P* < 0.001 versus FE*p*-Tyr; ^§^ *P* = 0.006 vs. FE*m*-Tyr.

APACHE II, acute physiology and chronic health evaluation II; MODS, multiple organ dysfunction score; SAPS II, simplified acute physiology score II; DID, daily insulin dose; IGP, insulin-glucose product; FE, fractional excretion.

Data expressed as mean ± SD or median (interquartile range).

**Table 2:** Predictors of carbohydrate metabolism parameters among septic patients, across the whole study period.

	DID		IGP^*^	
	*β*	*P*	*β*	*P*
Serum Phe	−0.450	0.001	−0.460	0.001
Serum *p*-Tyr/Phe	0.507	<0.001	0.554	<0.001
Serum *o*-Tyr/Phe	—	—	0.280	0.049

Urinary *m*-Tyr	0.381	0.007	0.382	0.007
Urinary *m*-Tyr/*p*-Tyr	0.359	0.011	0.351	0.013
Urinary *o*-Tyr/*p*-Tyr	0.322	0.023	0.308	0.030
Urinary (*m*-Tyr+*o*-Tyr)/*p*-Tyr	0.389	0.006	0.376	0.008

Model: body weight, hsCRP, PCT, daily hydrocortisone dose, daily dobutamine dose, and the actual amino acid parameter.

DID, daily insulin dose; IGP, insulin-glucose product.

^*^Calculated by average daily glucose level (mmol/L) multiplied by daily insulin dose (U).

Method: stepwise.

## References

[1] Stumvoll M., Chintalapudi U., Perriello G., Welle S., Gutierrez O., Gerich J. (1995). Uptake and release of glucose by the human kidney: postabsorptive rates and responses to epinephrine. *The Journal of Clinical Investigation*.

[2] Meyer C., Nadkarni V., Stumvoll M., Gerich J. (1997). Human kidney free fatty acid and glucose uptake: evidence for a renal glucose-fatty acid cycle. *American Journal of Physiology: Endocrinology and Metabolism*.

[3] Davidson M. B., Peters A. L. (1997). An overview of metformin in the treatment of type 2 diabetes mellitus. *American Journal of Medicine*.

[4] Meyer C., Stumvoll M., Nadkarni V., Dostou J., Mitrakou A., Gerich J. (1998). Abnormal renal and hepatic glucose metabolism in type 2 diabetes mellitus. *The Journal of Clinical Investigation*.

[5] Meyer C., Stumvoll M., Welle S., Woerle H. J., Haymond M., Gerich J. (2003). Relative importance of liver, kidney, and substrates in epinephrine-induced increased gluconeogenesis in humans. *American Journal of Physiology: Endocrinology and Metabolism*.

[6] Meyer C., Dostou J., Nadkarni V., Gerich J. (1998). Effects of physiological hyperinsulinemia on systemic, renal and hepatic substrate metabolism. *The American Journal of Physiology—Renal Physiology*.

[7] Cersosimo E., Garlick P., Ferretti J. (1999). Insulin regulation of renal glucose metabolism in humans. *American Journal of Physiology—Endocrinology and Metabolism*.

[8] Wang Y., DeMayo J. L., Hahn T. M. (1992). Tissue- and development-specific expression of the human phenylalanine hydroxylase/chloramphenicol acetyltransferase fusion gene in transgenic mice. *The Journal of Biological Chemistry*.

[9] Rao D. N., Kaufman S. (1986). Purification and state of activation of rat kidney phenylalanine hydroxylase. *Journal of Biological Chemistry*.

[10] Crimi E., Sica V., Slutsky A. S. (2006). Role of oxidative stress in experimental sepsis and multisystem organ dysfunction. *Free Radical Research*.

[11] Mühl D., Woth G., Drenkovics L. (2011). Comparison of oxidative stress & leukocyte activation in patients with severe sepsis & burn injury. *Indian Journal of Medical Research*.

[12] Ware L. B., Fessel J. P., May A. K., Roberts L. J. (2011). Plasma biomarkers of oxidant stress and development of organ failure in severe sepsis. *Shock*.

[13] Stadtman E. R., Berlett B. S. (1991). Fenton chemistry: amino acid oxidation. *The Journal of Biological Chemistry*.

[14] Galano A., Cruz-Torres A. (2008). OH radical reactions with phenylalanine in free and peptide forms. *Organic and Biomolecular Chemistry*.

[15] Ayling J. E., Pirson W. D., Al-Janabi J. M., Helfand G. D. (1974). Kidney phenylalanine hydroxylase from man and rat. Comparison with the liver enzyme. *Biochemistry*.

[16] Lubec B., Hayn M., Denk W., Bauer G. (1996). Brain lipid peroxidation and hydroxy radical attack following the intravenous infusion of hydrogen peroxide in an infant. *Free Radical Biology and Medicine*.

[17] Dandona P., Mohanty P., Hamouda W. (2001). Rapid communication: inhibitory effect of a two day fast on reactive oxygen species (ROS) generation by leucocytes and plasma ortho-tyrosine and meta-tyrosine concentrations. *Journal of Clinical Endocrinology and Metabolism*.

[18] Jörres R. A., Holz O., Zachgo W. (2000). The effect of repeated ozone exposures on inflammatory markers in bronchoalveolar lavage fluid and mucosal biopsies. *The American Journal of Respiratory and Critical Care Medicine*.

[19] Molnár G. A., Wagner Z., Markó L. (2005). Urinary ortho-tyrosine excretion in diabetes mellitus and renal failure: evidence for hydroxyl radical production. *Kidney International*.

[20] Toth P., Koller A., Pusch G. (2011). Microalbuminuria, indicated by total versus immunoreactive urinary albumins, in acute ischemic stroke patients. *Journal of Stroke and Cerebrovascular Diseases*.

[21] Molnár G. A., Nemes V., Biró Z., Ludány A., Wagner Z., Wittmann I. (2005). Accumulation of the hydroxyl free radical markers meta-, ortho-tyrosine and DOPA in cataractous lenses is accompanied by a lower protein and phenylalanine content of the water-soluble phase. *Free Radical Research*.

[22] Szijártó I. A., Molnár G. A., Mikolás E. (2014). Increase in insulin-induced relaxation of consecutive arterial segments toward the periphery: role of vascular oxidative state. *Free Radical Research*.

[23] Kun S., Mikolás E., Molnár G. A. (2014). Association of plasma ortho-tyrosine/para-tyrosine ratio with responsiveness of erythropoiesis-stimulating agent in dialyzed patients. *Redox Report*.

[24] Mikolás E., Kun S., Laczy B. (2014). Incorporation of ortho- and meta-tyrosine into cellular proteins leads to erythropoietin-resistance in an erythroid cell line. *Kidney & Blood Pressure Research*.

[25] Levy M. M., Fink M. P., Marshall J. C. (2003). 2001 SCCM/ESICM/ACCP/ATS/SIS international sepsis definitions conference. *Critical Care Medicine*.

[26] Dellinger R. P., Levy M. M., Rhodes A. (2013). Surviving sepsis campaign: international guidelines for management of severe sepsis and septic shock 2012. *Intensive Care Medicine*.

[27] Würtz P., Soininen P., Kangas A. J. (2013). Branched-chain and aromatic amino acids are predictors of insulin resistance in young adults. *Diabetes Care*.

